# Prevalence and influencing factors of lower urinary tract symptoms in female nurses: a cross-sectional study based on TARGET

**DOI:** 10.3389/fpubh.2023.1201184

**Published:** 2023-06-19

**Authors:** Xinyue Zhang, Mengli Li, Wenshuo Dong, Xiaoyan Lv, Li Li, Xiaorong Yang, Yingjuan Cao

**Affiliations:** ^1^School of Nursing and Rehabilitation, Shandong University, Jinan, Shandong, China; ^2^Department of Nursing, Qilu Hospital of Shandong University, Shandong, Jinan, China; ^3^Nursing Theory and Practice Innovation Research Center, Shandong University, Jinan, Shandong, China; ^4^Clinical Epidemiology Unit, Qilu Hospital of Shandong University, Jinan, Shandong, China

**Keywords:** lower urinary tract symptoms, female nurses, risk factors, prevalence, cross-sectional study

## Abstract

**Background:**

Even though occupational women have a high incidence of lower urinary tract symptoms (LUTS), which seriously affect their daily work life, few large scale sample studies have provided empirical evidence to support this phenomenon among female nurses in China. Consequently, this article investigated female nurses who was presupposed to have a high prevalence of LUTS, which adversely exposes their health and patient safety to these risks. Additionally, it is considered important to explore the factors associated with LUTS in female nurses for patient care safety and nurse bladder health practice.

**Objectives:**

The purpose of this study was to assess the incidence of LUTS and symptoms-related risk factors among female nurses, to provide evidence for the prevention and control of LUTS.

**Methods:**

An online survey recruiting 23,066 participants was carried out in a multicenter cross-sectional study in 42 hospitals from December 2020 to November 2022. Stepwise multivariate logistic regression analysis and nomogram were used to identify the factors associated with lower urinary tract symptoms. Besides, SPSS version 26.0, R version 4.2.2, and GraphPad Prism Version 8.3 software packages were used for statistical analysis.

**Results:**

Based on the completion rate of the questionnaire which was 84.1% (n = 19,393), it was found that among 19,393 female nurses, the prevalence of LUTS was 67.71% and this rate was influenced by age, Body Mass Index (BMI), marital status, years of working, menstrual status, mode of delivery, history of breastfeeding, history of miscarriage, history of alcohol and coffee or tea consumption (*p* < 0.05). Interestingly, we also find that in addition to the above mentioned factors, anxiety, depression, and perceived stress were also related to LUTS in female nurses (*p* < 0.05).

**Conclusion:**

Given the high prevalence of LUTS among female nurses and their potential influencing factors, female nurses should focus on their reproductive health and develop good lifestyle habits. Thus, nursing managers should provide a warm and harmonious work environment and sensitize female nurses to increase their awareness about the importance of drinking clean water and urinating during work in a hygienic environment.

## 1. Introduction

Lower urinary tract symptoms (LUTS) have been defined by the International Continence Society (ICS) as three primary symptom groups: storage, voiding, and postmicturition symptoms (1). Unambiguously, based on the findings of numerous scientific and empirical studies, women are more susceptible to LUTS than men—due to the anatomy and physiology of the female genitourinary system (2). Similarly, women’s hormonal environmental experiences and gender-centered social experiences during pregnancy and childbirth (3), can be associated with this health issue (4–6). LUTS have greatly and adversely impacted women’s physical, mental, and sexual health (7) and also reducing their quality of life (8). Thus, LUTS unwittingly impact women’s productivity at work (9), as well as lead to social isolation and shame, which concomitantly increases the risk of depression and anxiety (10), in addition to elevating the financial burden caused by treatment (11).

That said, the findings of some previous studies have indicated that there was a bi-directional relationship between LUTS and work (12). On top of that, since nurses comprise the largest proportion of healthcare occupations throughout the world, with women making up at least 90 percent of this occupational group (13, 14), investigating the effect of LUTS on their work patterns is important. Over and beyond, heavy workloads, long shifts, insufficient breaks, delayed voiding, and limited fluid intake are common unhealthy behaviors affecting nurses’ bladders (15), which epitomizes a global nursing occupational problem (16). Correspondingly, this recognizable characteristic of the nurses’ profession is an issue that contributes to their vulnerability to LUTS (17), so it attracts the extensive attention of researchers.

Furthermore, given that the incidence of LUTS in the female nursing workforce is considerably higher than that in the general population (18, 19), it is an issue of great concern to health practitioners globally. The prevalence of at least one type of LUTS is estimated to be 43% (20) to 97% (2) among female nurses. Apart from that, China’s huge population of over 1.4 billion, evokes some critical issues of diversity in the LUTS faced by female nurses generating an unstable development trend, and diseases related factors are complicated and diverse (21). Numerous studies have shown female nurses with LUTS have been diagnosed to often have co-occurring psychological, physical, and social needs (17, 22–27). Analogously, the effect of work-related factors on LUTS has been generally ignored (2), accentuating the impact of years of working, shift patterns, fluid intake during work, etc., on the severity of this scourge on health-related work productivity is vital. Evidentially, LUTS is therefore more common in female nurses when compared to other medical staff populations (18). Consequently, it is essential to study the influence factors in this diseased population to shed more light on the condition of LUTS among female nurses.

Contemporaneously, the identification of individual, behavioral, and environmental associated with LUTS, as well as occupational factors among female nurses might considerably increase the level of awareness of the importance of preventing lower urinary tract symptoms in nurses. Hence, this study aims to assess the prevalence of LUTS and symptom-related risk factors among female nurses in China based on TARGET (28) study, to provide substantial evidence for the prevention and control of LUTS nationwide.

## 2. Methods and material

### 2.1. Design, setting, and participants

In this study, the baseline data elicited from the Nurses’ Health Cohort Study of Shandong [registration number: ChiCTR2100043202] conducted between December 2020 to November 2022 was used. In addition, the dataset was organized using a multi-center cross-sectional study technique and data were collected from nurses in 42 hospitals and 16 cities in the Shandong Province of China. The inclusion criteria included all registered nurses with nursing qualification certificates who volunteered to participate in the study within the enumeration areas. More so, the exclusion criteria were comprised of [a] retired, refresher, and student nurses, [b] nurses who suffered from severe mental illness or took psychotropic drugs; [c] nurses who had been working for <6 months; and [d] nurses who were on leave during the investigation.

### 2.2. Measures

The questionnaire contained socio-demographic information, International Consultation on Incontinence Questionnaire-Female Lower Urinary Tract Symptoms, ICIQ-FLUTS [Chinese version], Generalized Anxiety Disorder-7 [GAD-7], Patient Health Questionnaire-9 [PHQ-9], and Perceived Stress Scale [PSS], which were distributed among Chinese female nurses.

Additionally, demographic variables included: [1] Age, educational background, marital status, and Body Mass Index (BMI);[2] Work-related variables such as professional title, monthly income, working years, shift mode, and shift working years; [3] Living habits variables which encompassed smoking, drinking frequency, drinking coffee or tea during work, fluid intake during work; and [4] Female health variables such as regular menstruation, pregnant times, parturition times, delivery mode, lactation history, as well as abortion history.

The LUTS of female nurses was measured by the Chinese version of the International Consultation on Incontinence Questionnaire-Female Lower Urinary Tract Symptoms, ICIQ-FLUTS, which was translated by Huang et al. (29). The questionnaire included 12 items in 4 dimensions, including urinary storage symptoms, voiding symptoms, incontinence symptoms and the degree of influence of each symptom on daily life. The 5-level scoring method was used: never, occasionally, sometimes, most of the time, and always. The higher the score, the more serious the symptoms. According to the definition of lower urinary tract symptoms by the International Continence Society in 2012 and the specific items of the ICIQ-FLUTS scale, this study classified the symptoms of storage period [nocturia, urinary urgency, bladder discomfort, frequent urination], urination period [urination hesitation, urination difficulty, urinary flow interruption], incontinence symptoms [urgent urinary incontinence, frequent urinary leakage, stress urinary incontinence, enuresis, nocturnal enuresis] as LUTS, that is, the subjects with any one of the above symptoms can be diagnosed as LUTS (30). The LUTS distress score is 0 for no distress, 1–4 for mild distress, 5–7 for moderate distress, and 8–10 for severe distress. If the research object chooses more than 1 point for any symptom, it is necessary to describe the degree of influence of symptoms on their daily life, and the score range is 0–10 [no influence-extreme influence]. The Cronbach’s α for the LUTS score in this study was 0.811, and the Cronbach’s α for the degree of symptom distress was 0.954.

The Generalized Anxiety Disorder 7-Item Scale [GAD-7], designed by Spitzer et al., with a pretest reliability of 0.830 and a Cronbach’s α of 0.920, indicates good reliability and validity, and has been widely used to screen for the presence and severity of anxiety (31). The total GAD-7 score ranges from 0 to 21 points scoring from zero [not at all] to three (nearly every day) such that 0–4, 5–9, 10–14, and 15–21 scores represent normal, mild, moderate, and severe anxiety, respectively (31). He et al. (32) validated the reliability and validity of the Chinese version of the GAD-7, the test–retest reliability was 0.856 and the Cronbach’s α was 0.898. The Cronbach’s α of the GAD-7 was 0.962 in this study.

The Patient Health Questionnaire [PHQ-9] which contains nine items has been widely used to measure depression symptoms (33). Kroenke et al. examined the reliability and validity of the PHQ-9 and found the retest reliability to be 0.840 and the Cronbach’s α of the questionnaire to be 0.890. The total PHQ-9 score ranges from 0 to 27 points, scoring from zero [not at all] to three [nearly every day] such that 0–4, 5–9, 10–14, 15–19, and 20–27 points represent normal, mild, moderate, moderately severe, and severe depression, respectively (34). Wang et al. validated the reliability and validity of the Chinese version of the PHQ-9 (35), the test–retest reliability was 0.860 and the Cronbach’s α was 0.860. The Cronbach’s α of the PHQ-9 was 0.954 in this study.

The perceived stress scale which contains 10 items was used to assess nurses’ perceptions of stress (36). The Chinese version of the perceived pressure scale used in this study has been verified to have good reliability and validity (37). The coefficient alpha values for the positive and negative subscales were 0.83 and 0.76 for PSS-10, respectively. Each item is measured on a 5-point Likert scale from 0 [never] to 4 [always], and the perceived stress level was divided into three categories: low [0–13], medium [14–27], and high [28–40]. The total PSS-10 score ranges from 0–40 points scoring from zero [never] to four [always] such that 0–13, 14–27, and 28–40 points represent low [0–13], medium [14–27], and high [28–40] perceived stress, respectively. The Cronbach’s α of the scale in this study was 0.932.

### 2.3. Data collection

Nurses who met the inclusion criteria and agreed to volunteer to enroll in the study, after signing an informed consent form, were duly recruited to complete the questionnaire. The recruitment of all the respondents was carried out through a combination of online and on-site recruitment methods. Online recruitment was carried out *via* the dissemination of recruitment information through the WeChat instant messaging and social media platform, hospital website, and text messaging. However, on-site recruitment was carried out through presentations, seminars, and *via* the use of recruitment posters in collaboration with the selected hospitals’ senior administrators such as directors and/or chief nurses of the nursing department of the participating hospital (28).

Thanks to the support of the Shandong Provincial Health and Wellness Commission and the National Institute of Health Care Data, the research team signed a research project cooperation agreement with the participating hospitals and obtained informed consent from each participant. In this online questionnaire, participants first registered their personal information using the official WeChat account of the Shandong Nurses Health Cohort Study. Then, they completed an electronic questionnaire sent by the official WeChat account. The survey questionnaire took about 10 to 15 min to complete. During the data collection process, the research team set up a separate cloud server and a MySQL database cluster (28).

Data managers have access to the data platform of the Nurse Health Cohort Study through an authentication mechanism for data interface calls, data maintenance, and data status monitoring. Only professionals who have signed a confidentiality agreement have direct access to this data. To ensure data quality, the questions and data logic control design required for electronic questionnaires were used (28).

### 2.4. Data analysis

The descriptive statistics were used to assess continuous variables [i.e., mean (M) ± standard deviation (SD)] and categorical variables [such as frequencies and percentages (%)]. One-way analysis of variance (ANOVA) was performed to examine the difference in continuous variables, and the significance of the difference in categorical variables was assessed *via* chi-squared testing. Furthermore, variables that were statistically significant in the univariate analysis were considered for inclusion in the backward stepwise multivariate logistic regression analysis to identify the main factors associated with LUTS, and to estimate the adjusted odds ratio (AOR) and 95% confidence interval (CI). Thereafter, the final logistic regression model was determined by the Akaike Information Criterion (AIC) minimization, and variables in the final model were indicated as statistically significant differences at *p* < 0.05.

The independent influence variables screened by logistic regression analysis were imported into the software. The “rms package” in R version 4.2.2 was used to build the nomogram model, and the receiver-operating characteristic (ROC) curves, calibration curves and Precision-Recall (Pr) curves were also plotted to evaluate the prediction capability of the model capability. Other data analyses were performed using the SPSS version 26.0. Drawing forest map with GraphPad Prism Version 8.3. The level of significance was set at *p* < 0.05.

## 3. Results

### 3.1. Prevalence and characteristics of LUTS in female nurses

**Figure 1:**
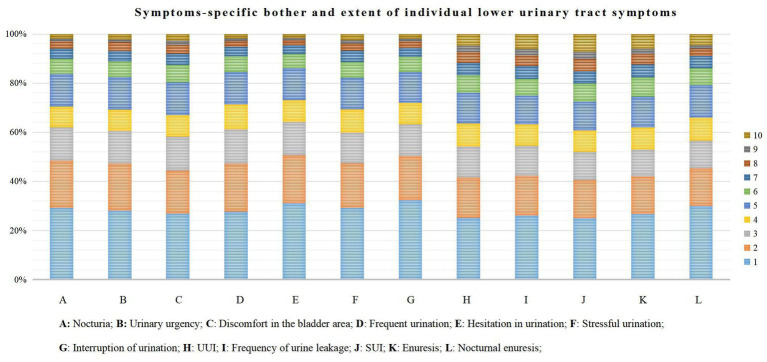
Symptoms-specific bother and extent of individual lower urinary tract symptoms. **(A)** Nocturia; **(B)** Urinary urgency; **(C)** Discomfort in the bladder area; **(D)** Frequent urination; **(E)** hesitation in urination; **(F)** Stressful urination; **(G)** Interruption of urination; **(H)** UUI; **(I)** Frequency of urine leakage; **(J)** SUI; **(K)** Enuresis; **(L)** Normal enuresis.

Table 1 shows the distribution of LUTS symptoms among female nurses and the impact on daily life. A total of 23,066 nurses clicked on the questionnaire web link, and 19,393 female nurses completed the questionnaire, indicating an overall response rate of 84.1%. It was observed that the prevalence of LUTS among participating female nurses was high at approximately 67.71%. While, the prevalence of storage symptoms, urination symptoms, and incontinence symptoms were 32.15, 12.95, and 56.80%, respectively. Notably, the prevalence of stress urinary incontinence (SUI) was the highest at 50.47%, just as the probability of frequent urination is lowest at 1.62%. Similarly, incontinence symptoms had the greatest degree of impact on the daily life of female nurses, with 1,444 (i.e., 15.32%) being severely influenced by stress urinary incontinence (SUI) and 1,028 (i.e., 13.12%) being severely affected by leakage of urine (Figure 1).

**Table 1:** Distribution of symptoms of LUTS and its impact on daily life among female nurses.

Symptoms	*N* (%)	ICIQ-FLUTS			
		Impact of daily life (M ± SD)	Degree stratification/N (%)		
			Mild	Moderate	Severe
*Urinary storage symptoms*	6,235 (32.15%)	6.33 ± 8.12	—	—	—
Nocturia	3,209 (16.55%)	1.66 ± 2.31	6,913 (70.33%)	2,312 (23.52%)	605 (6.15%)
Urinary urgency	3,326 (17.15%)	1.77 ± 2.39	7,006 (69.02%)	2,428 (23.92%)	716 (7.05%)
Bladder discomfort	1,534 (7.91%)	1.45 ± 2.33	5,327 (66.86%)	1991 (24.99%)	649 (8.15%)
Frequent urination	315 (1.62%)	1.46 ± 2.20	6,107 (71.06%)	2024 (23.55%)	463 (5.39%)
*Voiding symptoms*	2,511 (12.95%)	3.36 ± 5.74	—	—	—
Hesitation in urination	1,543 (7.96%)	1.17 ± 2.02	5,283 (73.01%)	1,612 (22.28%)	341 (4.71%)
Stressful urination	902 (4.65%)	1.15 ± 2.11	4,569 (69.28%)	1,572 (23.84%)	454 (6.88%)
Interruption of urination	1,047 (5.40%)	1.05 ± 1.99	4,586 (71.87%)	1,430 (22.41%)	365 (5.72%)
*Incontinence symptoms*	11,016 (56.80%)	7.08 ± 10.92	—	—	—
UUI	7,973 (41.13%)	1.64 ± 2.56	5,293 (63.52%)	2054 (24.65%)	986 (11.83%)
Frequency of urine leakage	7,313 (37.71%)	1.56 ± 2.58	4,936 (63.02%)	1869 (23.86%)	1,028 (13.12%)
SUI	9,788 (50.47%)	1.96 ± 2.82	5,723 (60.70%)	2,261 (23.98%)	1,444 (15.32%)
Enuresis	3,515 (18.13%)	1.14 ± 2.30	3,549 (62.02%)	1,461(25.53%)	712 (12.44%)
Nocturnal enuresis	1,498 (7.72%)	0.78 ± 1.91	2,797 (65.75%)	1,074 (25.25%)	383 (9.00%)

(*N* = 19,393).

UUI, urgent urinary incontinence; SUI, stress urinary incontinence.

Table 2 shows that the mean age of the 19,393 participants was 34.64 (SD = 7.095) years. While the mean working years for nurses was 26.65 (SD = 7.495) years. Likewise, the mean BMI of nurses was 22.59 (SD = 3.152). Approximately 91.4% of participants had bachelor’s degrees, 79.0% were married, and 50.5% had a primary title. Additionally, approximately half of the participants with an average monthly income of less than or equal to 6,000 Chinese Yuan (CNY). Interestingly, over half of the participants (i.e., 63.3%) have been working in shifts, while nearly half (i.e., 46.3%) had worked in shifts for more than 5 years.

**Table 2:** Descriptive statistics and univariate analysis of variables related to lower urinary tract symptoms in female nurses.

Characteristics	Total	LUTS, *n* (%)		*χ* ^2^ */F*	*p* value
		No	Yes		
Personal traits					
Age				3.873	**0.049**
	34.64 ± 7.095	32.70 ± 6.859	35.56 ± 7.019		
BMI				16.251	**<0.001**
	22.59 ± 3.152	21.99 ± 3.033	22.88 ± 3.168		
Education				83.397	**<0.001**
Secondary vocational degree	46 (0.2%)	14 (0.2%)	32 (0.2%)		
Associate’s degree	1,321 (6.8%)	576 (9.2%)	745 (5.7%)		
Bachelor’s degree	17,724 (91.4%)	5,582 (89.1%)	12,142 (92.5%)		
Master’s degree	302 (1.6%)	90 (1.4%)	212 (1.6%)		
Marital status				974.392	**<0.001**
Unmarried	3,636 (18.7%)	1,964 (31.4%)	1,672 (12.7%)		
Married	15,317 (79.0%)	4,155 (66.4%)	11,162 (85.0%)		
Widowhood/divorce	356 (1.8%)	122 (1.9%)	234 (1.8%)		
Others	84 (0.4%)	21 (0.3%)	63 (0.5%)		
Work-related factors					
Years of working				748.582	**<0.001**
	26.65 ± 7.495	24.82 ± 6.105	27.52 ± 7.926		
Professional title				495.497	**<0.001**
Primary	9,794 (50.5%)	3,883 (62.0%)	5,911 (45.0%)		
Medium	8,173 (42.1%)	2,063 (32.9%)	6,110 (46.5%)		
Senior	1,409 (7.3%)	311 (5.0%)	1,098 (8.4%)		
Others	17 (0.1%)	5 (0.1%)	12 (0.1%)		
Monthly income/CNY				160.286	**<0.001**
<3,000	1,287 (6.6%)	552 (8.8%)	735 (5.6%)		
3,000–6,000	9,663 (49.8%)	3,342 (53.4%)	6,321 (48.1%)		
6,001–9,000	5,845 (30.1%)	1,652 (26.4%)	4,193 (31.9%)		
9,001–1,2000	1,841 (9.5%)	497 (7.9%)	1,344 (10.2%)		
>1,2000	757 (3.9%)	219 (3.5%)	538 (4.1%)		
Scheduling mode				32.440	**<0.001**
Day shift	7,006 (36.1%)	2,087 (33.3%)	4,919 (37.5%)		
Small night shift	29 (0.1%)	11 (0.2%)	18 (0.1%)		
Big night shift	78 (0.4%)	30 (0.5%)	48 (0.4%)		
Work in shifts	12,280 (63.3%)	4,134 (66.0%)	8,146 (62.0%)		
Shift work experience/Years					
0–5 years	10,418 (53.7%)	3,757 (60.2%)	6,651(50.7%)	215.237	**<0.001**
6–10 years	4,691 (24.2%)	1,463 (23.4%)	3,228 (24.6%)		
11–15 years	3,157 (16.3%)	802 (12.8%)	2,355 (17.9%)		
16–20 years	842 (4.3%)	170 (2.7%)	672 (5.1%)		
>20 years	285 (1.5%)	60 (1.0%)	225 (1.7%)		
Female physiological factor					
Menstrual regularity				168.909	**<0.001**
Extremely regular	3,075 (15.9%)	1,226 (19.6%)	1,849 (14.1%)		
Very regular	6,113 (31.5%)	2,038 (32.5%)	4,075 (31.0%)		
Regular	4,177 (21.5%)	1,303 (20.8%)	2,874 (21.9%)		
Most of the time earlier than expected	877 (4.5%)	251 (4.0%)	626 (4.8%)		
Most of the time later than expected	1817 (9.4%)	573 (9.2%)	11,244 (9.5%)		
Uncertain, sooner or later	1,651 (8.5%)	490 (7.8%)	1,161 (8.8%)		
No menstruation	1,683 (8.7%)	381 (6.1%)	1,302 (9.9%)		
Number of Gravidity				957.574	**<0.001**
0	5,720 (29.5%)	2,719 (43.4%)	3,001 (22.9%)		
1 ~ 2	9,322 (48.1%)	2,664 (42.5%)	6,658 (50.7%)		
≥3	4,351 (22.4%)	879 (14.0%)	3,472 (26.4%)		
Number of production				859.609	**<0.001**
0	6,147 (31.7%)	2,873 (45.9%)	3,274 (24.9%)		
1 ~ 2	13,076 (67.4%)	3,349 (53.5%)	9,727 (74.1%)		
≥3	170 (0.9%)	40 (0.6%)	130 (1.0%)		
Obstetric mode of delivery				993.096	**<0.001**
Vaginal delivery	7,897 (40.7%)	1715 (27.4%)	6,182 (47.1%)		
Cesarean section	5,349 (27.6%)	1,674 (26.7%)	3,675 (28.0%)		
Not delivered	6,147 (31.7%)	2,873 (45.9%)	3,274 (24.9%)		
History of lactation				888.534	**<0.001**
No	6,789 (35.0%)	3,118 (49.8%)	3,671 (28.0%)		
Yes	12,604 (65.0%)	3,144 (50.2%)	9,460 (72.0%)		
History of abortion				469.642	**<0.001**
No	13,196 (68.0%)	4,919 (78.6%)	8,277 (63.0%)		
Yes	6,197 (32.0%)	1,343 (21.4%)	4,854 (37.0%)		
Life behavioral factors					
Smoking				4.034	0.401
Never smoke	19,332 (99.7%)	6,243 (99.7%)	13,089 (99.7%)		
Used to smoke (Cumulative ≤5 packs)	35 (0.2%)	12 (0.2%)	23 (0.2%)		
Used to smoke(Cumulative >5 packs)	7 (0.0%)	0 (0.0%)	7 (0.1%)		
Still smoking now(Cumulative≤5 packs)	9 (0.0%)	4 (0.1%)	5 (0.0%)		
Still smoking now(Cumulative>5 packs)	10 (0.1%)	3 (0.0%)	7 (0.1%)		
Drinking				123.465	**<0.001**
Never	14,861 (76.6%)	5,103 (81.5%)	9,758 (74.3%)		
Occasionally	4,133 (21.3%)	1,067 (17.0%)	3,066 (23.3%)		
Sometimes	350 (1.8%)	82 (1.3%)	268 (2.0%)		
Often	49 (0.3%)	10 (0.2%)	39 (0.3%)		
Coffee or tea				79.470	**<0.001**
No	13,027 (67.2%)	4,479 (71.5%)	8,548 (65.1%)		
Yes	6,366 (32.8%)	1,783 (28.5%)	4,583 (34.9%)		
Liquid intake during work				9.200	0.101
<500 mL	9,704 (50.0%)	3,054 (48.8%)	6,650 (50.6%)		
500-1,000 mL	7,375 (38.0%)	2,432 (38.8%)	4,943 (37.6%)		
1,001-1,500 mL	1,477 (7.6%)	491 (7.8%)	986 (7.5%)		
1,501-2000 mL	601 (3.1%)	198 (3.2%)	403 (3.1%)		
2001-2,500 mL	153 (0.8%)	52 (0.8%)	101 (0.8%)		
>2,500 mL	83 (0.4%)	35 (0.6%)	48 (0.4%)		
Psychological factors					
Anxiety rating				780.340	**<0.001**
Normal	8,638 (44.5%)	3,657 (58.4%)	4,981 (37.9%)		
Mild	7,873 (40.6%)	2,075 (33.1%)	5,798 (44.2%)		
Moderate	1,942 (10.0%)	361 (5.8%)	1,581 (12.0%)		
Severe	940 (4.8%)	169 (2.7%)	771 (5.9%)		
Depression rating				876.467	**<0.001**
Normal	8,149 (42.0%)	3,537 (56.5%)	4,612 (35.1%)		
Mild	7,661 (39.5%)	2,065 (33.0%)	5,596 (42.6%)		
Moderate	1,643 (8.5%)	301 (4.8%)	1,342 (10.2%)		
Moderately severe	1,297 (6.7%)	250 (4.0%)	1,047 (8.0%)		
Severe	643 (3.3%)	109 (1.7%)	534 (4.1%)		
Perceived stress rating				204.584	**<0.001**
Low	1,468 (7.6%)	565 (9.0%)	903 (6.9%)		
Moderate	16,362 (84.4%)	5,435 (86.8%)	10,927 (83.2%)		
High	1,563 (8.1%)	262 (4.2%)	1,301 (9.9%)		

Bold value for *p* < 0.05. (*N* = 19,393).

When considering women’s health, it was found that approximately one-third of participants experienced irregular menstruation. Over and above that, about 70% of them have the experience of gravidity, with 68.3% having given birth at least once, 40.7% having a history of vaginal delivery, 65.0% having a history of lactation, and just as 32.0% had a history of abortion. Moreover, it should be noted that 99.7% of participants reported never smoking, 76.6% reported never drinking alcohol, and 32.8% reported drinking coffee or tea, while half of the participants reported consuming less than 500 mL of fluid during work.

Furthermore, with regards to mental health, it was found that nurses with LUTS (*n* = 13,131), 62.1% (*n* = 8,150) experienced some level of anxiety (where, mild = 44.2%, moderate = 12.0%, severe = 5.9%), while 65.1% (*n* = 8,620) experienced some level of depression (where, mild = 42.6%, moderate =10.2%, moderately severe = 8.0%, severe = 4.1%), and 93.2% (*n* = 12,329) experienced some level of perceived stress (where, medium = 83.2%, high = 9.9%).

### 3.2. The univariate analysis of factors associated with LUTS

Table 2 also shows all the respondents’ univariate analysis of LUTS from work-related characteristics, female health characteristics, dietary characteristics, and mental health characteristics. It was thereafter established that there were significant differences in distribution patterns between the group with LUTS and the group without it (*p* < 0.05). It was uncovered that these differences can be grouped by respondents’ age, education, marital status, BMI, professional title, monthly income, years of work, scheduling mode, shift work experience, menstrual regularity, gravidity, production, obstetric mode of delivery, history of lactation, history of abortion, drinking alcohol, consuming coffee or tea, as well as anxiety, depression, and perceived stress rating levels.

### 3.3. Stepwise multivariate logistic regression analysis of factors associated with LUTS

**Figure 2:**
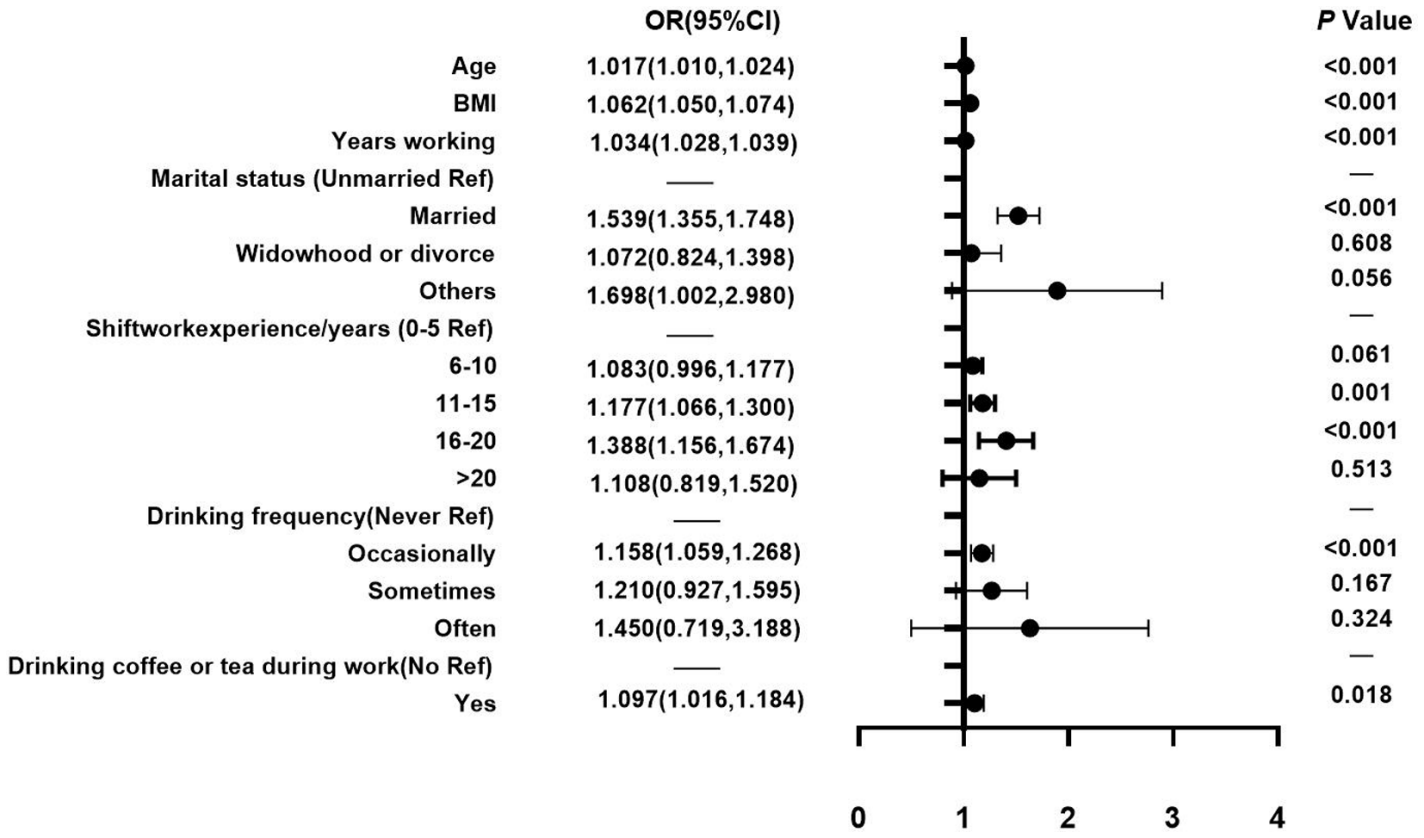
Demographic and work-related factors.

Table 3 shows the results of the backward stepwise multivariate logistic regression model with an adjusted odds ratio (AOR) and 95% CI and the main variables including demographic factors, work-related factors, living habits factors, female health factors, and psychological factors. With regards to demographic and work-related factors (Figure 2), it was found that among female nurses, each 1-unit increase in BMI was associated with a 6.2% increase in the odds of female nurses suffering from LUTS in this study. Relatedly, the odds of having LUTS increased by either 1.7% or 3.4% for every 1-year increase in age or working years among female nurses, respectively. Comparatively, when unmarried nurses were compared to those who were married, nurses who were married were significantly more likely to experience LUTS (AOR = 1.539, 95% CI: 1.355–1.748). Besides, relative to nurses who had 0–5 years of shift work experience, those who had between 11–15 years and 16–20 years were significantly more likely to experience LUTS than those at the early stage of their career (AOR = 1.177, 95% CI: 1.066–1.300; AOR = 1.388, 95% CI: 1.156–1.674). With regards to living habits (Figure 2), it was found that when nurses who never drank alcohol were compared to those who drink occasionally, those who drank alcohol occasionally (AOR =1.158, 95% CI: 1.059–1.268) were significantly more likely to experience LUTS. Nevertheless, drinking coffee or tea was also associated with LUTS among female nurses (AOR = 1.097, 95% CI: 1.016–1.184).

**Table 3:** Backward stepwise multivariate logistic regression analysis of factors associated with lower urinary tract symptoms in female nurses.

Variables	Adjusted OR	95% CI	*p* value
Personal traits			
Age	1.017	(1.010–1.024)	**<0.001**
BMI	1.062	(1.050–1.074)	**<0.001**
Marital status			
Unmarried	1.00 (Ref.)		
Married	1.539	(1.355–1.748)	**<0.001**
Widowhood/divorce	1.072	(0.824–1.398)	0.608
Others	1.698	(1.002–2.980)	0.056
Work-related factors			
Years of working	1.034	(1.028–1.039)	**<0.001**
Shift work experience/years			
0–5	1.00 (Ref.)		
6–10	1.083	(0.996–1.177)	0.061
11–15	1.177	(1.066–1.300)	**0.001**
16–20	1.388	(1.156–1.674)	**<0.001**
>20	1.108	(0.819–1.520)	0.513
Female physiological factors			
Menstrual regularity			
Extremely regular very regular	1.00 (Ref.)		
Very Regular	1.269	(1.151–1.398)	**<0.001**
Regular	1.405	(1.264–1.562)	**<0.001**
Most of the time earlier than expected	1.374	(1.153–1.641)	**<0.001**
Most of the time later than expected	1.343	(1.176–1.535)	**<0.001**
Uncertain, sooner or later	1.467	(1.276–1.687)	**<0.001**
No menstruation	2.178	(1.854–2.561)	**<0.001**
Gravidity/frequency			
0	1.00 (Ref.)		
1–2	0.870	(0.686–1.106)	0.253
>2	1.096	(0.831–1.446)	0.518
Obstetric mode of delivery			
Vaginal delivery	1.00 (Ref.)		
Cesarean section	0.572	(0.526–0.622)	**<0.001**
Not delivered	0.647	(0.513–0.819)	**<0.001**
History of lactation			
No	1.00 (Ref.)		
Yes	1.534	(1.321–1.779)	**<0.001**
History of abortion			
No	1.00 (Ref.)		
Yes	1.144	(1.029–1.273)	**0.013**
Life behavioral factors			
Drinking			
Never	1.00 (Ref.)		
Occasionally	1.158	(1.059–1.268)	**<0.001**
Sometimes	1.210	(0.927–1.595)	0.167
often	1.450	(0.719–3.188)	0.324
Coffee or tea			
No	1.00 (Ref.)		
Yes	1.097	(1.016–1.184)	**0.018**
Psychological factors			
Anxiety rating			
Normal	1.00 (Ref.)		
Mild	1.345	(1.223–1.480)	**<0.001**
Moderate	1.677	(1.406–2.004)	**<0.001**
Severe	1.303	(0.995–1.714)	0.056
Depression rating			
Normal	1.00 (Ref.)		
Mild	1.548	(1.406–1.704)	**<0.001**
Moderate	2.069	(1.751–2.451)	**<0.001**
Moderately severe	1.829	(1.488–2.252)	**<0.001**
Severe	2.050	(1.470–2.866)	**<0.001**
Perceived stress rating			
Low	1.00 (Ref.)		
Moderate	0.971	(0.858–1.099)	0.644
High	1.254	(1.007–1.565)	**0.044**

Bold value for *p* < 0.05. (*N* = 16,262).

**Figure 3:**
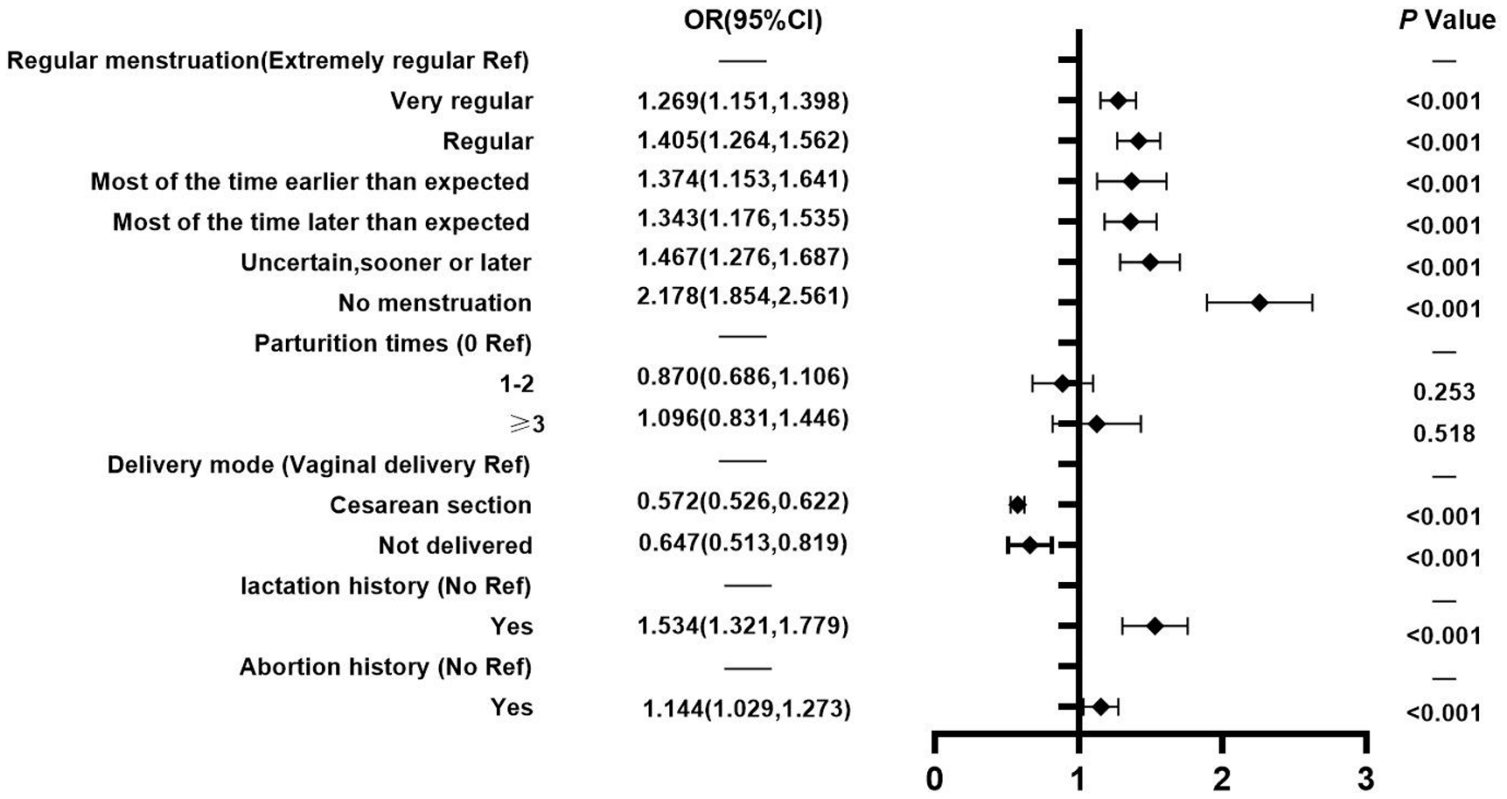
Female health factors.

In terms of female health factors (Figure 3), nurses whose menstruation was very regular, regular, and most of the time earlier than expected, as well as those whose menstruation was most of the time later than expected, uncertain, sooner or later, and with no menstruation were significantly more likely to experience LUTS than those who had extremely regular menstruation (AOR = 1.269, 95% CI: 1.151–1.398; AOR = 1.405, 95% CI: 1.264–1.562; AOR = 1.374, 95% CI: 1.153–1.641; AOR = 1.343, 95% CI: 1.176–1.535; AOR = 1.467, 95% CI: 1.276–1.687; and AOR = 2.178, 95% CI:1.854–2.561), respectively. Additionally, female nurses who experienced lactation (AOR =1.534, 95% CI: 1.321–1.779) or experienced abortion (AOR = 1.144, 95% CI: 1.029–1.273) were more likely to develop LUTS than nurses who did not have such experience. Interestingly, nurses who went through cesarean section delivery or have not delivered (AOR = 0.572, 95% CI: 0.526–0.622; AOR = 0.647, 95% CI: 0.513–0.819) were less likely to develop LUTS than those who had a vaginal delivery.

**Figure 4:**
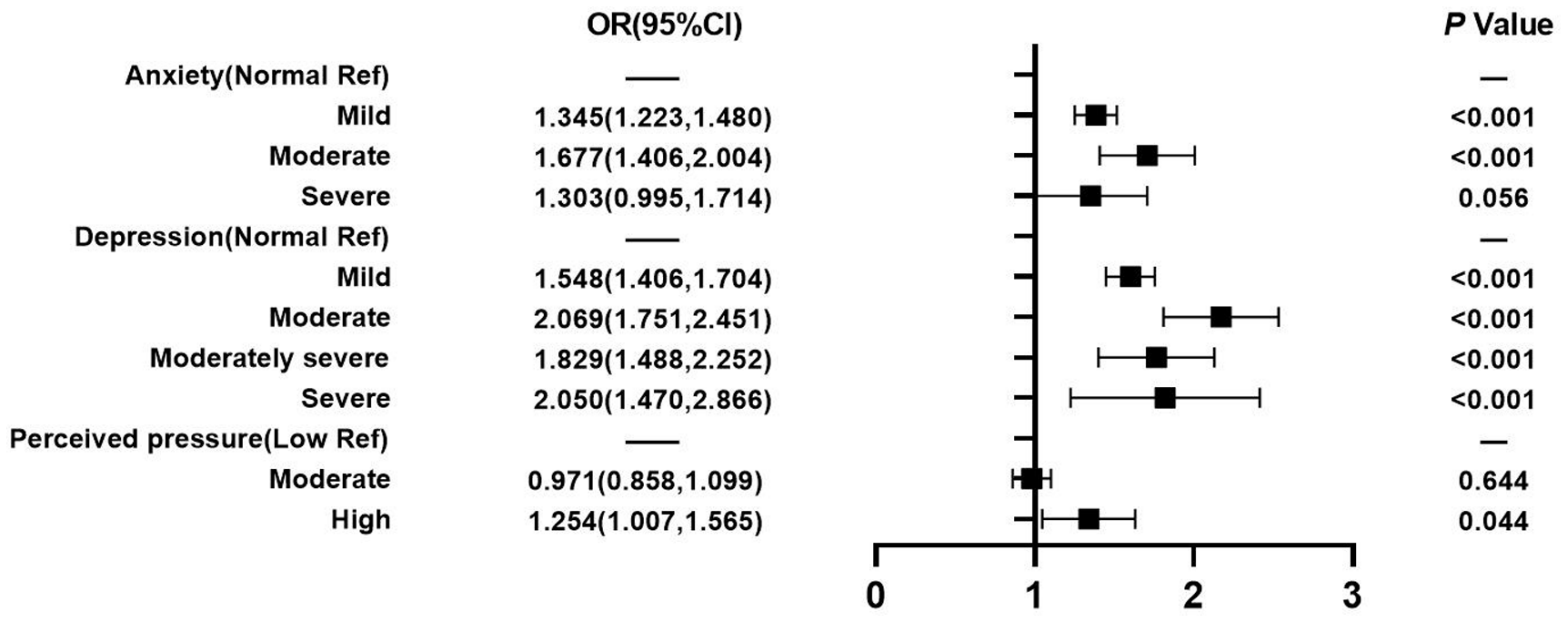
Mental health factors.

Figure 4 reveals that mental health factors were also found to be associated with LUTS. When nurses who reported a normal level of anxiety were compared to those who reported a mild or moderate level of anxiety, nurses who reported mild or moderate levels of anxiety were significantly more likely to experience LUTS, with an AOR of 1.345 (95% CI: 1.223–1.480) and AOR of 1.677 (95% CI: 1.406–2.004), respectively. Similarly, nurses who reported mild, moderate, moderately severe, or severe levels of depression were significantly more likely to experience LUTS, with AOR of 1.548 (95% CI: 1.406–1.704), 2.069 (95% CI: 1.751–2.451), 1.829 (95% CI: 1.488–2.252), and 2.050 (95% CI: 1.470–2.866), respectively. Likewise, a perceived high-stress level had a statistically significant association with LUTS when compared to a perceived normal-stress level, with an AOR of 1.254 (95% CI: 1.007–1.565).

### 3.4. Nomogram model of LUTS in female nurses

**Figure 5:**
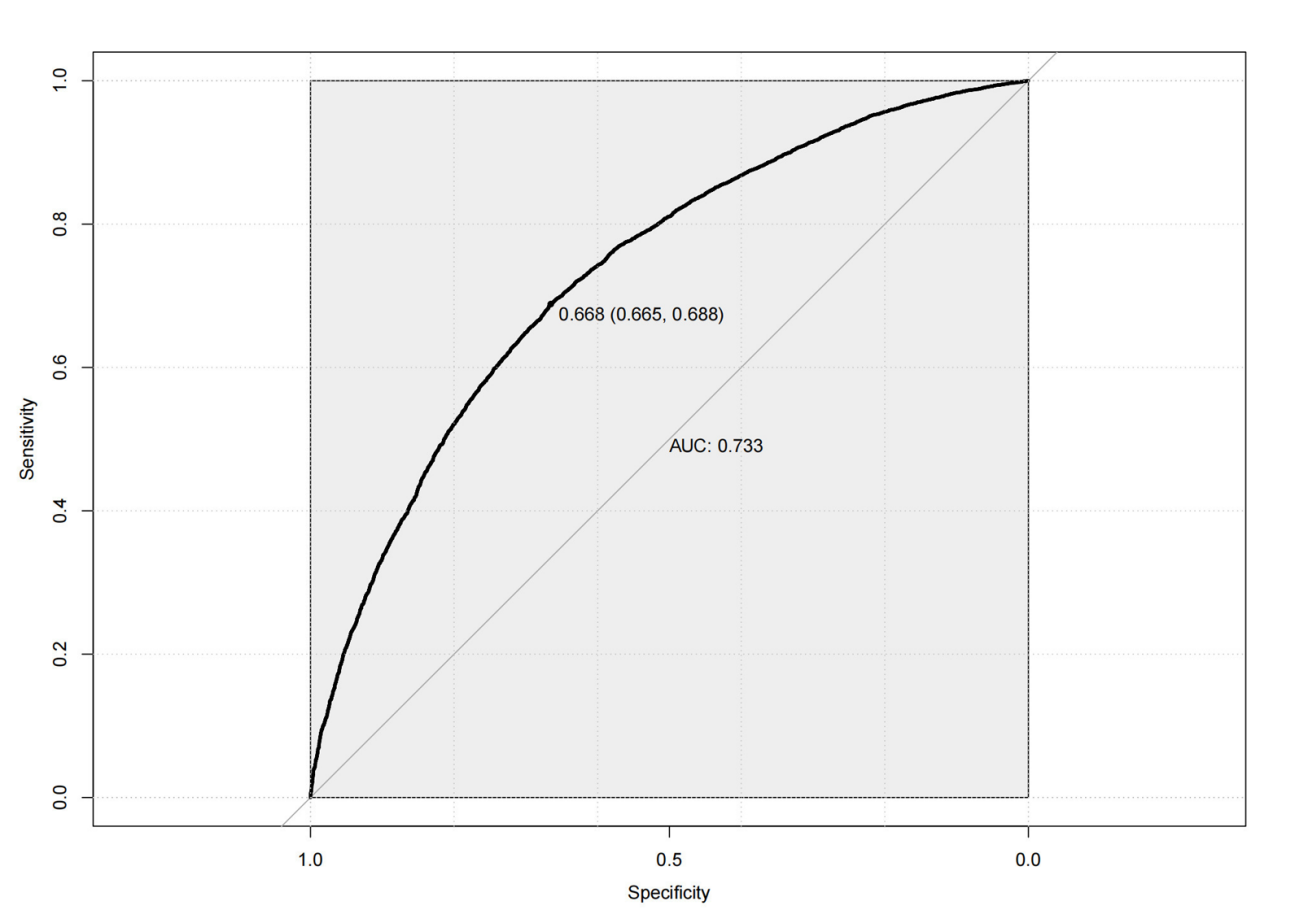
The receiver operating characteristic (ROC) curves.

**Figure 6:**
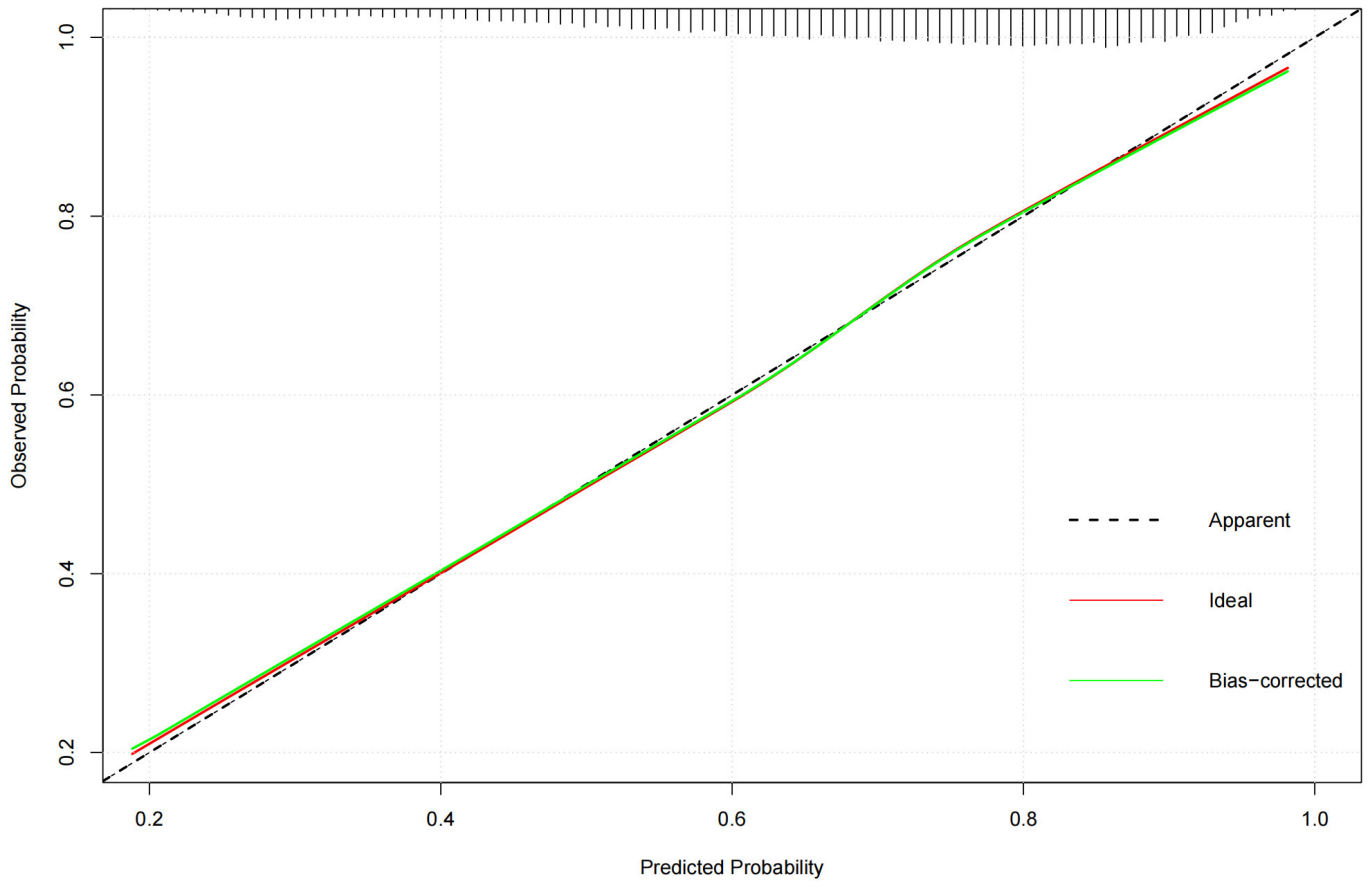
The calibration curves.

**Figure 7:**
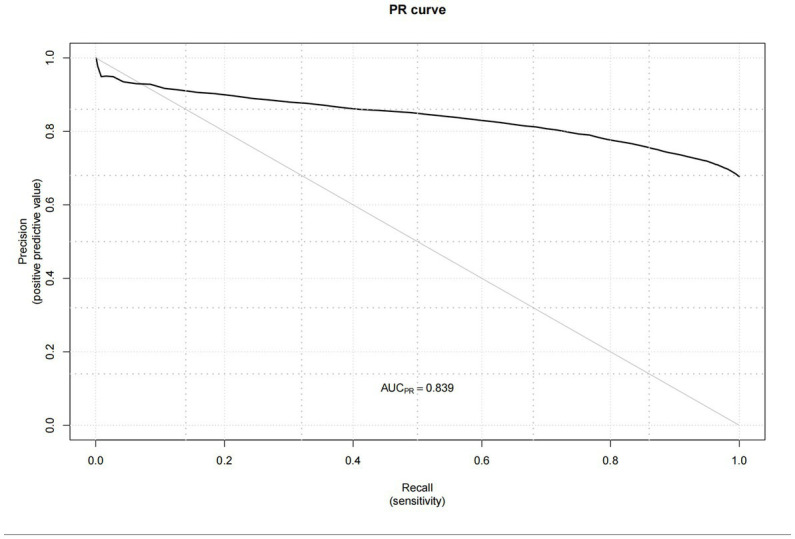
The Precision-Recall (Pr) curves.

**Figure 8:**
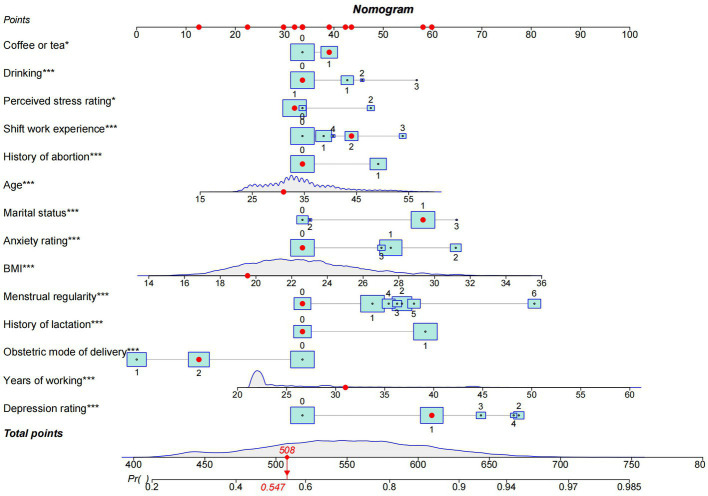
The nomogram model.

Next, the performance of the model empirically was evaluated before analyzing its predictors. The results indicated in Figure 5 showed that the predictive efficiency of the logistic regression model was adequate (sensitivity = 0.688, specificity = 0.665, accuracy = 0.681, and area under the receiver operating characteristic curve [AUROC] = 0.733 (95% CI: 0.725–0.740)). The calibration curves (Figure 6) were close to the ideal prediction with a mean absolute error (MAE) of 0.005 after 1,000 bootstrap self-sampling. Furthermore, The Precision-Recall (Pr) curves were depicted in Figure 7. Consequently, the predictive performance of the models that were adopted in this study was guaranteed. The column line graph model (Figure 8) was constructed using 14 risk predictors, which can personalize the calculation of the score corresponding to each independent influencing factor, as well as count the total score and show the predicted value corresponding to the total score which can predict the probability of occurrence of LUTS.

## 4. Discussion

### 4.1. Prevalence of LUTS in female nurses

The study was conducted to assess the prevalence and risk factors of LUTS among Chinese female nurses. The result of this study showed that the prevalence of LUTS among 19,393 female nurses from Shandong, China was 67.71%. This estimate is higher than the rate of prevalence of LUTS in Turkey (20), which stands at 44.5%, but lower than the reported 97.36% prevalence rate of LUTS in Iran (2), in comparison to the 89.63% prevalence rate of LUTS among female nurses in Beijing, China (38). An analysis of data obtained from the pilot study carried out by The Taiwan Nurse Bladder Survey (TNBS) revealed that there was a 65% prevalence rate of LUTS among female nurses in Taipei (17), Taiwan which is roughly consistent with the current findings (67.71%). However, this figure is significantly higher than the prevalence rate of LUTS among the adult female population in China, which stands at 55.5% (39). This result indicated that female nurses might considerably suffer from the impact of LUTS in China. Moreover, increased LUTS prevalence could lead to reduced work efficiency or even unemployment, as well as affect their lifelong physical and mental health later in life. Accordingly, the inconsistencies emanating from the different prevalence rates of LUTS in similar studies can be linked to the time frame of prior studies, the research and analysis methods employed, as well as the diverse composition of the studied population, which can be limited to the entire population, age group, and/or ethnicity (40). Therefore, it is hoped that a large scale epidemiological survey of female nurses will be conducted in all countries, using the same questionnaire, to reveal the bladder health status of female nurses worldwide.

### 4.2. Personal traits and work-related factors

Age and BMI are well-known risk factors influencing the sufferers of LUTS. This assumption is reinforced by the findings of the present study. That said, it has been demonstrated in extant studies carried out across North America (41) and Asia (25, 42). Nonetheless, overweight or obese women with long-term abdominal hypertension may experience an increase in bladder pressure and urethral mobility, which can lead to pelvic organ prolapse, and an increased burden on the pelvic floor muscles (43). In addition, constipation might also increase abdominal pressure during physical activity or defecation, which in turn leads to LUTS (24). Due to a combination of factors and reasons, constipation-related issues were not included in this study. Hence, it was hoped that related studies will include this variable in future research centered on LUTS. In a study by Yuliang et al. in China (44) and a systematic review by Sedighe et al. worldwide (24), bare that excessive BMI is a negative factor in increasing the incidence of LUTS among female nurses. Over and beyond, marriage is also a critical risk factor affecting LUTS among female nurses in China, when this segment of the population is compared to unmarried women, which is consistent with the findings of similar studies (25, 45). Physiologically, married women begin to experience sexual behavior, pregnancies, and even deliveries, and they are easily exposed to LUTS. Psychologically, married women have to take on the burden of a family and deal with complex interpersonal issues, therefore, aggravating stressful conditions may possibly increase the chance of LUTS.

In terms of work-related factors, years of working and long shift working were significantly associated with LUTS among female nurses. Similarly, Zhang’s study (46), revealed that there was a statistically significant difference between work experience and the probability of LUTS among female nurses. The reason for this phenomenon might be the heavy workload, in addition to the high level of work stress experienced over the years by these nurses, which may negatively affect their bladder function. It was also assumed that since the years of work are proportional to age, the relationship between years of work and LUTS is not hard to understand. Additionally, common work environmental factors associated with occupational stress such as high work demand and work overload negatively impacted female nurses’ exposure to LUTS in China (47). As Hyoungseob et al. discovered that unhealthy toileting behaviors to empty their bladders, which mediates the relationship between occupational stress and OAB (48), and the most common behavior was delayed urination. This Phenomenon illustrates the gravity of stress-related issues affecting female nurses in China.

### 4.3. Life behavioral factors

In this study, drinking alcohol occasionally increased the risk of female nurses contracting LUTS when compared to those that never attempted to do so. However, drinking alcohol sometimes or often drinking alcohol is not associated with LUTS among female nurses. Notably, similar results have been reported in previous studies, although the findings of these studies were controversial (25, 49). Apart from that, light alcohol consumption induces LUTS by increasing sympathetic nervous system activity thereby exerting diuretic effects on the antidiuretic hormone system (50), while moderate to a high level of alcohol consumption appears to reduce the prevalence of LUTS due to enhanced estrogen receptor induction (51). Nevertheless, the frequency of smoking was empirically proven to be unrelated to LUTS, thereby debunking what was put forward by past studies in this area (27, 44). Interestingly, this finding is possibly because the population included in this study was a group of female nurses with relatively healthy lifestyles.

What’s more, it is found that coffee or tea consumption is a risk factor for LUTS among female nurses in China, possibly mediated by caffeine effects such as an increase in diuresis, central nervous stimulation, and increased contractility of the lower urinary tract smooth muscle. Yet, this finding remains disputed, since there is no association between coffee consumption and LUTS found in several cross-sectional studies (52, 53). Interestingly, in Tomlinson’s study, there is an association between a reduction in dietary caffeine intake and a reduction in daytime incontinence, although this was not proven to be statistically significant in this research (54). Additionally, a recent pilot study of 11 women showed an overall reduction in bladder symptoms with caffeine-free beverages when compared to women that ingested caffeine-containing beverages (55). In like manner, the relationship between tea intake and LUTS was substantiated in one study which established a positive association between women who drank two or more cups of tea per day and overactive bladder and nocturia, just as there exists a negative association between tea consumption and stress urinary incontinence (56). The caffeine content of tea is about one-third of that found in coffee (57), suggesting that tea may contain components other than caffeine that may also be related to lower urinary tract dysfunction.

### 4.4. Female physiological factors

The related analysis of physiological factors revealed that female nurses were prone to risk factors that trigger LUTS such as irregular menstruation, mode of vaginal delivery, history of miscarriage, and history of breastfeeding. Apart from that, it has been scientifically proven that vaginal delivery and the number of deliveries were among the most important risk factors affecting female patients with urinary incontinence (58). However, based on the findings of an Indian study, there was no association between delivery and urinary incontinence, which is inconsistent with the findings of this study (59). Interestingly, hormonal effects and mechanical changes are considered to be the main causes of LUTS during pregnancy (60). In this study, it was found that a dose–response effect of pregnancy was critical, just as multiple pregnancies were an important risk factor for the development of LUTS, which may be the result of repetitive injury to the peripheral pelvic nerves, as well as direct injury to the pelvic supporting tissues (61). Similarly, Zhang et al. reported that women who underwent vaginal delivery were more likely to experience symptoms during the urine storage period (62). It is observed that this may be related to peripheral pelvic nerve injury and direct injury to the intrapelvic fascia. However, cesarean delivery can eliminate damage to either the pelvic floor muscle tissue or the public nerves during delivery, as well as prevent the development of LUTS among female nurses (62).

### 4.5. Psychological factors

In the current study, it has been proven that negative emotions including anxiety, depression, and perceived pressure contribute to the development of LUTS among female nurses, which has been reinforced by the findings of similar studies (63, 64). It was also envisaged that there is a link between psychological dysfunction and bladder function. Researchers have suggested that the pro-adrenocorticotropic hormone-releasing factor is expressed in areas of the central nervous system that control voiding and stress responses, while the pro-adrenocorticotropic hormone-releasing factor increases in response to anxiety, depression, pain, and dysfunction of the pelvic organs (65). We can therefore decisively submit that LUTS are not fatal but do have a serious impact on women’s mental health. LUTS can largely interfere with an individual’s self-image (66), consequently, the stigma and embarrassment caused by LUTS may lead to feelings of anxiety, depression, and stress among female nurses in China. This can be linked to the fact that patients with LUTS might worry about its development, the possibility of cancer, and their sexuality, which can ultimately create a certain amount of stress among this segment of the population (67).

### 4.6. Limitations

To the best of our knowledge, this is one of the largest sample studies on the prevalence of LUTS among female nurses in China. The large sample size and wide survey area are the main strengths of this study. Besides, this study involved hospitals at various levels across all the regions of Shandong Province of China which can better reflect the reality of the problems. However, the participants who did not meet the study requirements were excluded to obtain more realistic results. This study also has some limitations. First, the survey of the study gathering phase was carried out only within Shandong province, therefore, the results obtained cannot be extended to the whole country or even a larger area due to regional restrictions and differences. Second, it is a cross-sectional study and cannot verify the causal relationship therein, which implies that a large sample longitudinal study is required to ascertain the causal relationship that exists between factors and LUTS among female nurses in China. Third, due to occupational specificity, fewer male nurses, and the differences in the way LUTS are scored, no male group was included in this study, which is a major limitation when measuring the influence of gender on the infection rates of LUTS patients. Fourth, this study did not use objective indicators to measure LUTS, since the diagnosis we relied upon was based on scales rather than clinical criteria, thus, it shows the existence of recall bias in current dataset.

## 5. Conclusion

In summary, many of the risk factors associated with LUTS can be offset by a healthy lifestyle, proper urinary habits, frequent physical activity, and weight control (68). Consequently, for female nursing staff, attention needs to be paid to the impact of their work environment on their overall well-being, such as raising awareness among managers to provide educational opportunities for nurses, as well as advocating for the justification of the benefits of nurses’ personal health and bladder health practices in the workplace. Sadly, the habit among nurses of not drinking water or using the toilet in the workplace frequently is a hidden nursing workforce crisis that raises the issue of dignity and human rights in the healthcare industry. Managers, therefore, need to assess nurses’ needs and workplace barriers to healthy practices to ensure their water and toilet use is sufficient, as well as minimize barriers to work and maximize nurses’ bladder health *ceteris paribus* (69).

## Data availability statement

The datasets presented in this article are not readily available because the data from the Nurses’ Health Cohort Study of Shandong needs time for data clearing and the establishment of guidelines. This data will be made public in the future. Requests to access the datasets should be directed to caoyj@sdu.edu.cn.

## Ethics statement

The studies involving human participants were reviewed and approved by the Ethics Committee of Scientific Research of Shandong University Qilu Hospital. The patients/participants provided their written informed consent to participate in this study.

## Author contributions

XZ and ML: methodology, formal analysis, data curation, software, writing-original draft, and visualization. XZ, WD, and XL: writing-review and editing and project administration. XZ, ML, XL, and LL: investigation. XY: methodology. YC: conceptualization, resources, supervision, project administration, and funding acquisition. All authors contributed to the article and approved the submitted version.

## Conflict of interest

The authors declare that the research was conducted in the absence of any commercial or financial relationships that could be construed as a potential conflict of interest.

## Publisher’s note

All claims expressed in this article are solely those of the authors and do not necessarily represent those of their affiliated organizations, or those of the publisher, the editors and the reviewers. Any product that may be evaluated in this article, or claim that may be made by its manufacturer, is not guaranteed or endorsed by the publisher.

## References

[1] Haylen BT de Ridder D Freeman RM Swift SE Berghmans B Lee J. An international Urogynecological association (IUGA)/international continence society (ICS) joint report on the terminology for female pelvic floor dysfunction. Neurourol Urodyn. (2010) 29:4–20. doi: 10.1002/nau.20798, PMID: 19941278

[2] Nasiri M Sigaroudi AE Moghadamnia MT Leili EK. Lower urinary tract symptoms and related factors in Iranian female nurses. Iran J Nurs Midwifery Res. (2022) 27:280–6. doi: 10.4103/ijnmr.ijnmr_126_21, PMID: 36275339 PMC9580568

[3] Brady SS Bavendam TG Berry A Fok CS Gahagan S Goode PS. The prevention of lower urinary tract symptoms (PLUS) in girls and women: developing a conceptual framework for a prevention research agenda. Neurourol Urodyn. (2018) 37:2951–64. doi: 10.1002/nau.23787, PMID: 30136299 PMC6451314

[4] Maserejian NN Chen S Chiu GR Wager CG Kupelian V Araujo AB. Incidence of lower urinary tract symptoms in a population-based study of men and women. Urology. (2013) 82:560–4. doi: 10.1016/j.urology.2013.05.009, PMID: 23876577 PMC3758799

[5] Brady SS Berry A Camenga DR Fitzgerald CM Gahagan S Hardacker CT. Applying concepts of life course theory and life course epidemiology to the study of bladder health and lower urinary tract symptoms among girls and women. Neurourol Urodyn. (2020) 39:1185–02. doi: 10.1002/nau.24325, PMID: 32119156 PMC7659467

[6] Sutcliffe S Bavendam T Cain C Epperson CN Fitzgerald CM Gahagan S. The Spectrum of bladder health: the relationship between lower urinary tract symptoms and interference with activities. J Womens Health (Larchmt). (2019) 28:827–1. doi: 10.1089/jwh.2018.7364, PMID: 31058573 PMC6590721

[7] Coyne KS Sexton CC Kopp ZS Ebel-Bitoun C Milsom I Chapple C. The impact of overactive bladder on mental health, work productivity and health-related quality of life in the UK and Sweden: results from EpiLUTS. BJU Int. (2011) 108:1459–71. doi: 10.1111/j.1464-410X.2010.10013.x, PMID: 21371240

[8] Pizzol D Demurtas J Celotto S Maggi S Smith L Angiolelli G. Urinary incontinence and quality of life: a systematic review and meta-analysis. Aging Clin Exp Res. (2021) 33:25–35. doi: 10.1007/s40520-020-01712-y, PMID: 32964401 PMC7897623

[9] Markland A Chu H Epperson CN Nodora J Shoham D Smith A. Occupation and lower urinary tract symptoms in women: a rapid review and meta-analysis from the PLUS research consortium. Neurourol Urodyn. (2018) 37:2881–92. doi: 10.1002/nau.23806, PMID: 30272814 PMC6493329

[10] Choi EP Lam CL Chin WY. Mental health mediating the relationship between symptom severity and health-related quality of life in patients with lower urinary tract symptoms. Low Urin Tract Sympt. (2016) 8:141–9. doi: 10.1111/luts.12086, PMID: 27619778

[11] Powell LC Szabo SM Walker D Gooch K. The economic burden of overactive bladder in the United States: a systematic literature review. Neurourol Urodyn. (2018) 37:1241–9. doi: 10.1002/nau.23477, PMID: 29331047

[12] O'Shea SD Pope R Freire K Orr R. Prevalence of lower urinary tract symptoms in a cohort of Australian servicewomen and female veterans. Int Urogynecol J. (2022) 34:885–6. doi: 10.1007/s00192-022-05254-x 35763047 PMC10038961

[13] Australian Institute of Health and Welfare. Health workforce. Australian Institute of Health and Welfare. (2015) Available at: http://www.aihw.gov.au/workforce/ (accessed December 15, 2022).

[14] World Health Organization. Health workforce: nursing and mid-wifery personnel (2018). Global Health Observatory data) 2018

[15] Pierce H Perry L Gallagher R Chiarelli P. Culture, teams, and organizations: a qualitative exploration of female nurses' and midwives' experiences of urinary symptoms at work. J Adv Nurs. (2019) 75:1284–95. doi: 10.1111/jan.13951, PMID: 30644133

[16] Wendsche J Ghadiri A Bengsch A Wegge J. Antecedents and outcomes of nurses' rest break organization: a scoping review. Int J Nurs Stud. (2017) 75:65–80. doi: 10.1016/j.ijnurstu.2017.07.005, PMID: 28750245

[17] Liao YM Yang CY Kao CC Dougherty MC Lai YH Chang Y. Prevalence and impact on quality of life of lower urinary tract symptoms among a sample of employed women in Taipei: a questionnaire survey. Int J Nurs Stud. (2009) 46:633–4. doi: 10.1016/j.ijnurstu.2008.12.001, PMID: 19155010

[18] Pierce H Perry L Chiarelli P Gallagher R. A systematic review of prevalence and impact of symptoms of pelvic floor dysfunction in identified workforce groups. J Adv Nurs. (2016) 72:1718–34. doi: 10.1111/jan.12909, PMID: 26887537

[19] Wan X Wu C Xu D Huang L Wang K. Toileting behaviors and lower urinary tract symptoms among female nurses: a cross-sectional questionnaire survey. Int J Nurs Stud. (2017) 65:1–7. doi: 10.1016/j.ijnurstu.2016.10.005, PMID: 28027949

[20] Kaya Y Kaya C Baseskioglu B Ozerdoğan N Yenilmez A Demirüstü C. Effect of work-related factors on lower urinary tract symptoms in nurses and secretaries. Low Urin Tract Sympt. (2016) 8:49–54. doi: 10.1111/luts.12073, PMID: 26789543

[21] Qi M Hu X Liu J Wen J Hu X Wang Z. The impact of the COVID-19 pandemic on the prevalence and risk factors of workplace violence among healthcare workers in China. Front Public Health. (2022) 10:938423. doi: 10.3389/fpubh.2022.938423, PMID: 35958846 PMC9358256

[22] Xie X Chen Y Khan A Long T Li S Xie M. Risk factors for urinary incontinence in Chinese women: a cross-sectional survey. Female Pelvic Med Reconstr Surg. (2021) 27:377–1. doi: 10.1097/SPV.0000000000000871, PMID: 32282523

[23] Chuang YC Liu SP Lee KS Liao L Wang J Yoo TK. Prevalence of overactive bladder in China, Taiwan and South Korea: results from a cross-sectional, population-based study. Low Urin Tract Sympt. (2019) 11:48–55. doi: 10.1111/luts.12193, PMID: 28967230 PMC7379992

[24] Batmani S Jalali R Mohammadi M Bokaee S. Prevalence and factors related to urinary incontinence in older adults women worldwide: a comprehensive systematic review and meta-analysis of observational studies. BMC Geriatr. (2021) 21:212. doi: 10.1186/s12877-021-02135-8, PMID: 33781236 PMC8008630

[25] Wang Y Xu K Hu H Zhang X Wang X Na Y. Prevalence, risk factors, and impact on health related quality of life of overactive bladder in China. Neurourol Urodyn. (2011) 30:1448–55. doi: 10.1002/nau.21072, PMID: 21826714

[26] Vaughan CP Markland AD. Urinary incontinence in women. Ann Intern Med. (2020) 172:ITC17–32. doi: 10.7326/AITC202002040, PMID: 32016335

[27] Noh JW Yoo KB Kim KB Kim JH Kwon YD. Association between lower urinary tract symptoms and cigarette smoking or alcohol drinking. Transl Androl Urol. (2020) 9:312–1. doi: 10.21037/tau.2020.03.07, PMID: 32420137 PMC7214987

[28] Lv X Cao Y Li Y Liu Y Li R Guan X. The TARGET Nurses' health cohort study protocol: towards a revolution in getting nurses' health ticked. J Adv Nurs. (2022) 78:1815–23. doi: 10.1111/jan.15204, PMID: 35352386

[29] Huang L Zhang SW Wu SL Ma L Deng XH. The Chinese version of ICIQ: a useful tool in clinical practice and research on urinary incontinence. Neurourol Urodyn. (2008) 27:522–4. doi: 10.1002/nau.20546, PMID: 18351586

[30] Abrams P Cardozo L Fall M Griffiths D Rosier P Ulmsten U. The standardisation of terminology in lower urinary tract function: report from the standardisation sub-committee of the international continence society. Urology. (2003) 61:37–49. doi: 10.1016/S0090-4295(02)02243-4, PMID: 12559262

[31] Spitzer RL Kroenke K Williams JB Löwe B. A brief measure for assessing generalized anxiety disorder: the GAD-7. Arch Intern Med. (2006) 166:1092–7. doi: 10.1001/archinte.166.10.1092, PMID: 16717171

[32] He XY Li CB Qian J Cui HS Wu WY. Reliability and validity of a generalized anxiety disorder scale in general hospital outpatients. Arch Psychiatry. (2010) 22:200–3. doi: 10.3969/j.issn.1002-0829.2010.04.002

[33] Levis B Benedetti A Thombs BD. Accuracy of patient health Questionnaire-9 (PHQ-9) for screening to detect major depression: individual participant data meta-analysis. BMJ. (2019) 365:l1476. doi: 10.1136/bmj.l1476, PMID: 30967483 PMC6454318

[34] Kroenke K Spitzer RL Williams JB. The PHQ-9: validity of a brief depression severity measure. J Gen Intern Med. (2001) 16:606–3. doi: 10.1046/j.1525-1497.2001.016009606.x, PMID: 11556941 PMC1495268

[35] Wang W Bian Q Zhao Y Li X Wang W Du J. Reliability and validity of the Chinese version of the patient health questionnaire (PHQ-9) in the general population. Gen Hosp Psychiatry. (2014) 36:539–4. doi: 10.1016/j.genhosppsych.2014.05.021, PMID: 25023953

[36] Cohen S Kamarck T Mermelstein R. A global measure of perceived stress. J Health Soc Behav. (1983) 24:385–6. doi: 10.2307/2136404, PMID: 6668417

[37] Leung DY Lam TH Chan SS. Three versions of perceived stress scale: validation in a sample of Chinese cardiac patients who smoke. BMC Public Health. (2010) 10:513. doi: 10.1186/1471-2458-10-513 20735860 PMC2939644

[38] Chongjun H Chunfang Z Ting H Luping Y Qi W Bowen G. Prevalence of overactive bladder and other lower urinary tract symptoms in female nurses in Beijing and its association with occupational stress. Chin J Urol. (2008) 34:565–71. doi: 10.3760/cma.j.issn.1000-6702.2013.08.001

[39] Lei Z Lan Z Tao X Jinghe L Zhao L Gong J. A population-based epidemiology survey of the lower urinary tract symptoms in adult Chinese women: cross-sectional study. Beijing: Peking Union Medical College Hospital (PUMCH) (2015).

[40] Fan X Guo X Ren Z Li X He M Shi H. The prevalence of depressive symptoms and associated factors in middle-aged and elderly Chinese people. J Affect Disord. (2021) 293:222–8. doi: 10.1016/j.jad.2021.06.044, PMID: 34217959

[41] Stewart WF Van Rooyen JB Cundiff GW Abrams P Herzog AR Corey R. Prevalence and burden of overactive bladder in the United States. World J Urol. (2003) 20:327–6. doi: 10.1007/s00345-002-0301-4, PMID: 12811491

[42] Homma Y Yamaguchi O Hayashi K. An epidemiological survey of overactive bladder symptoms in Japan. BJU Int. (2005) 96:1314–8. doi: 10.1111/j.1464-410X.2005.05835.x, PMID: 16287452

[43] Wu XH Liu XX Xie KH Wang RM Wu YX Liu YG. Prevalence and related factors of urinary incontinence among Hebei women of China. Gynecol Obstet Investig. (2011) 71:262–7. doi: 10.1159/000320333, PMID: 21228537

[44] Wang Y Hu H Xu K Wang X Na Y Kang X. Prevalence, risk factors and the bother of lower urinary tract symptoms in China: a population-based survey. Int Urogynecol J. (2015) 26:911–9. doi: 10.1007/s00192-015-2626-8, PMID: 25653032

[45] Alshehri SZ Abumilha AK Amer KA Aldosari AA Shawkhan RA Alasmari KA. Patterns of urinary incontinence among women in Asir region. Saudi Arabia Cureus. (2022) 14:e21628. doi: 10.7759/cureus.21628, PMID: 35233309 PMC8881247

[46] Zhang C Hai T Yu L Liu S Li Q Zhang X. Association between occupational stress and risk of overactive bladder and other lower urinary tract symptoms: a cross-sectional study of female nurses in China. Neurourol Urodyn. (2013) 32:254–15. doi: 10.1002/nau.22290, PMID: 22903277

[47] Chang EM Hancock KM Johnson A Daly J Jackson D. Role stress in nurses: review of related factors and strategies for moving forward. Nurs Health Sci. (2005) 7:57–65. doi: 10.1111/j.1442-2018.2005.00221.x, PMID: 15670007

[48] Yoo H Kim JY Lee YM Kang MY. Occupational risk factors associated with lower urinary tract symptoms among female workers: a systematic review. Occup Environ Med. (2023) 80:288–6. doi: 10.1136/oemed-2022-108607, PMID: 36828632

[49] Joseph MA Harlow SD Wei JT Sarma AV Dunn RL Taylor JM. Risk factors for lower urinary tract symptoms in a population-based sample of African-American men. Am J Epidemiol. (2003) 157:906–4. doi: 10.1093/aje/kwg051, PMID: 12746243

[50] Hobson RM Maughan RJ. Hydration status and the diuretic action of a small dose of alcohol. Alcohol Alcohol. (2010) 45:366–3. doi: 10.1093/alcalc/agq029, PMID: 20497950

[51] Schatzl G Brössner C Schmid S Kugler W Roehrich M Treu T. Endocrine status in elderly men with lower urinary tract symptoms: correlation of age, hormonal status, and lower urinary tract function. Urology. (2000) 55:397–2. doi: 10.1016/S0090-4295(99)00473-2, PMID: 10699620

[52] Rortveit G Subak LL Thom DH Creasman JM Vittinghoff E Van Den Eeden SK. Urinary incontinence, fecal incontinence and pelvic organ prolapse in a population-based, racially diverse cohort: prevalence and risk factors. Female Pelvic Med Reconstr Surg. (2010) 16:278–3. doi: 10.1097/SPV.0b013e3181ed3e31, PMID: 22453506 PMC4976795

[53] Swithinbank L Hashim H Abrams P. The effect of fluid intake on urinary symptoms in women. J Urol. (2005) 174:187–9. doi: 10.1097/01.ju.0000162020.10447.31, PMID: 15947624

[54] Tomlinson BU Dougherty MC Pendergast JF Boyington AR Coffman MA Pickens SM. Dietary caffeine, fluid intake and urinary incontinence in older rural women. Int Urogynecol J Pelvic Floor Dysfunct. (1999) 10:22–8. doi: 10.1007/PL00004009, PMID: 10207763

[55] Wells MJ Jamieson K Markham TC Green SM Fader MJ. The effect of caffeinated versus decaffeinated drinks on overactive bladder: a double-blind, randomized, crossover study. J Wound Ostomy Continence Nurs. (2014) 41:371–8. doi: 10.1097/WON.0000000000000040, PMID: 24988515

[56] Tettamanti G Altman D Pedersen NL Bellocco R Milsom I Iliadou AN. Effects of coffee and tea consumption on urinary incontinence in female twins. BJOG. (2011) 118:806–3. doi: 10.1111/j.1471-0528.2011.02930.x, PMID: 21401855 PMC3094486

[57] Arya LA Myers DL Jackson ND. Dietary caffeine intake and the risk for detrusor instability: a case-control study. Obstet Gynecol. (2000) 96:85–9. doi: 10.1016/s0029-7844(00)00808-5, PMID: 10862848

[58] Hagan KA Erekson E Austin A Minassian VA Townsend MK Bynum JPW. A prospective study of the natural history of urinary incontinence in women. Am J Obstet Gynecol. (2018) 218:502.e1–8. doi: 10.1016/j.ajog.2018.01.045, PMID: 29425839 PMC5949886

[59] De Nunzio C Nacchia A Cicione A Cindolo L Gacci M Cancrini F. Physical activity as a protective factor for lower urinary tract symptoms in male patients: a prospective cohort analysis. Urology. (2019) 125:163–8. doi: 10.1016/j.urology.2018.12.035, PMID: 30634026

[60] Chaliha C Bland JM Monga A Stanton SL Sultan AH. Pregnancy and delivery: a urodynamic viewpoint. BJOG. (2000) 107:1354–9. doi: 10.1111/j.1471-0528.2000.tb11647.x, PMID: 11117761

[61] Snooks SJ Setchell M Swash M Henry MM. Injury to innervation of pelvic floor sphincter musculature in childbirth. Lancet. (1984) 2:546–15. doi: 10.1016/s0140-6736(84)90766-9, PMID: 6147604

[62] Zhang W Song Y He X Xu B Huang H He C. Prevalence and risk factors of lower urinary tract symptoms in Fuzhou Chinese women. Eur Urol. (2005) 48:309–3. doi: 10.1016/j.eururo.2005.03.003, PMID: 16005377

[63] Vakili M Amoqadiri M Mohammadi M Modaressi M. Prevalence and factors associated with stress urinary incontinence after childbirth of Yazd in 2015. J Shahid Sadoughi Univ Med Sci. (2016) 24:716–3.

[64] Milsom I Altman D Cartwright R Lapitan MC Nelson R Sillén U. Epidemiology of urinary incontinence (UI) and other lower urinary tract symptoms (LUTS), pelvic organ prolapse (POP) and anal incontinence (AI) In: Abrams P Cardozo L Khoury S Wein AJ, editors. Incontinence: 5th International Consultation on Incontinence. Paris: ICUD-EAU (2013)

[65] Klausner AP Steers WD. Corticotropin releasing factor: a mediator of emotional influences on bladder function. J Urol. (2004) 172:2570–3. doi: 10.1097/01.ju.0000144142.26242.f3, PMID: 15538210

[66] Gannon K Glover L O'Neill M Emberton M. Men and chronic illness: a qualitative study of LUTS. J Health Psychol. (2004) 9:411–15. doi: 10.1177/1359105304042350, PMID: 15117540

[67] Pinnock C O'Brien B Marshall VR. Older men's concerns about their urological health: a qualitative study. Aust N Z J Public Health. (1998) 22:368–3. doi: 10.1111/j.1467-842X.1998.tb01393.x, PMID: 9629824

[68] Liu B Wang L Huang SS Wu Q Wu DL. Prevalence and risk factors of urinary incontinence among Chinese women in Shanghai. Int J Clin Exp Med. (2014) 7:686–6. PMID: 24753764 PMC3992409

[69] Pierce HM Perry L Gallagher R Chiarelli P. Delaying voiding, limiting fluids, urinary symptoms, and work productivity: a survey of female nurses and midwives. J Adv Nurs. (2019) 75:2579–90. doi: 10.1111/jan.14128, PMID: 31236988

